# Refocusing the Use of Psychiatric Drugs for Treatment of Gastrointestinal Cancers

**DOI:** 10.3389/fonc.2020.01452

**Published:** 2020-08-14

**Authors:** Mariana Avendaño-Félix, Maribel Aguilar-Medina, Mercedes Bermudez, Erik Lizárraga-Verdugo, César López-Camarillo, Rosalío Ramos-Payán

**Affiliations:** ^1^Facultad de Ciencias Químico Biológicas, Universidad Autónoma de Sinaloa, Culiacán, Mexico; ^2^Posgrado en Ciencias Genómicas, Universidad Autónoma de la Ciudad de México, Mexico City, Mexico

**Keywords:** psychiatric drugs, cancer, esophageal, gastric, colorectal, hepatic, pancreatic

## Abstract

Gastrointestinal cancers (GICs) are the most common human tumors worldwide. Treatments have limited effects, and increasing global cancer burden makes it necessary to investigate alternative strategies such as drug repurposing. Interestingly, it has been found that psychiatric drugs (PDs) are promising as a new generation of cancer chemotherapies due to their anti-neoplastic properties. This review compiles the state of the art about how PDs have been redirected for cancer therapeutics in GICs. PDs, especially anti-psychotics, anti-depressants and anti-epileptic drugs, have shown effects on cell viability, cell growth, inhibition of proliferation (cell cycle arrest), apoptosis promotion by caspases activation or cytochrome C release, production of reactive oxygen species (ROS) and nuclear fragmentation over esophageal, gastric, colorectal, liver and pancreatic cancers. Additionally, PDs can inhibit neovascularization, invasion and metastasis in a dose-dependent manner. Moreover, they can induce chemosensibilization to 5-fluorouracil and cisplatin and can act synergistically with anti-neoplastic drugs such as gemcitabine, paclitaxel and oxaliplatin. All anti-cancer activities are given by activation or inhibition of pathways such as HDAC1/PTEN/Akt, EGFR/ErbB2/ErbB3, and PI3K/Akt; PI3K-AK-mTOR, HDAC1/PTEN/Akt; Wnt/β-catenin. Further investigations and clinical trials are needed to elucidate all molecular mechanisms involved on anti-cancer activities as well as adverse effects on patients.

## Introduction

Gastrointestinal cancers (GICs) are the most common human tumors worldwide, affecting esophageal, stomach, colorectum, liver, pancreas, and gallbladder tissues (1). The treatment for these malignancies is surgical resection, nonetheless, at the time of diagnosis, most of the cases are unresectable (2–4). Additionally, chemotherapy and radiotherapy show a modest improvement in survival. These limited results and the increasing global cancer burden require investigating new strategies such as drug repurposing.

Mental disorders and substance abuse are at present the leading cause of disability worldwide (5), giving as a result a wide use of psychiatric drugs (PDs) for treatment. These long-term drug therapies have been studied in order to elucidate if its use is a risk factor, but at the same time it is possible that their molecular mechanisms could be used to treat different cancer types. On the one hand, Food and Drug Administration (FDA) preclinical-limited to animal studies showed that 63.6% of tested anti-depressants, 90% of anti-psychotic, 70% of benzodiazepines and sedative-hypnotics, 25% of amphetamines and stimulants, and 85.7% of anti-convulsants were associated with carcinogenicity (6). On the other hand, it has been found that PDs are promising as a new generation of cancer chemotherapies due to their anti-neoplastic properties (7), such as autophagy, which can inhibits migration and invasiveness in different types of cancer (8).

**Figure 1:**
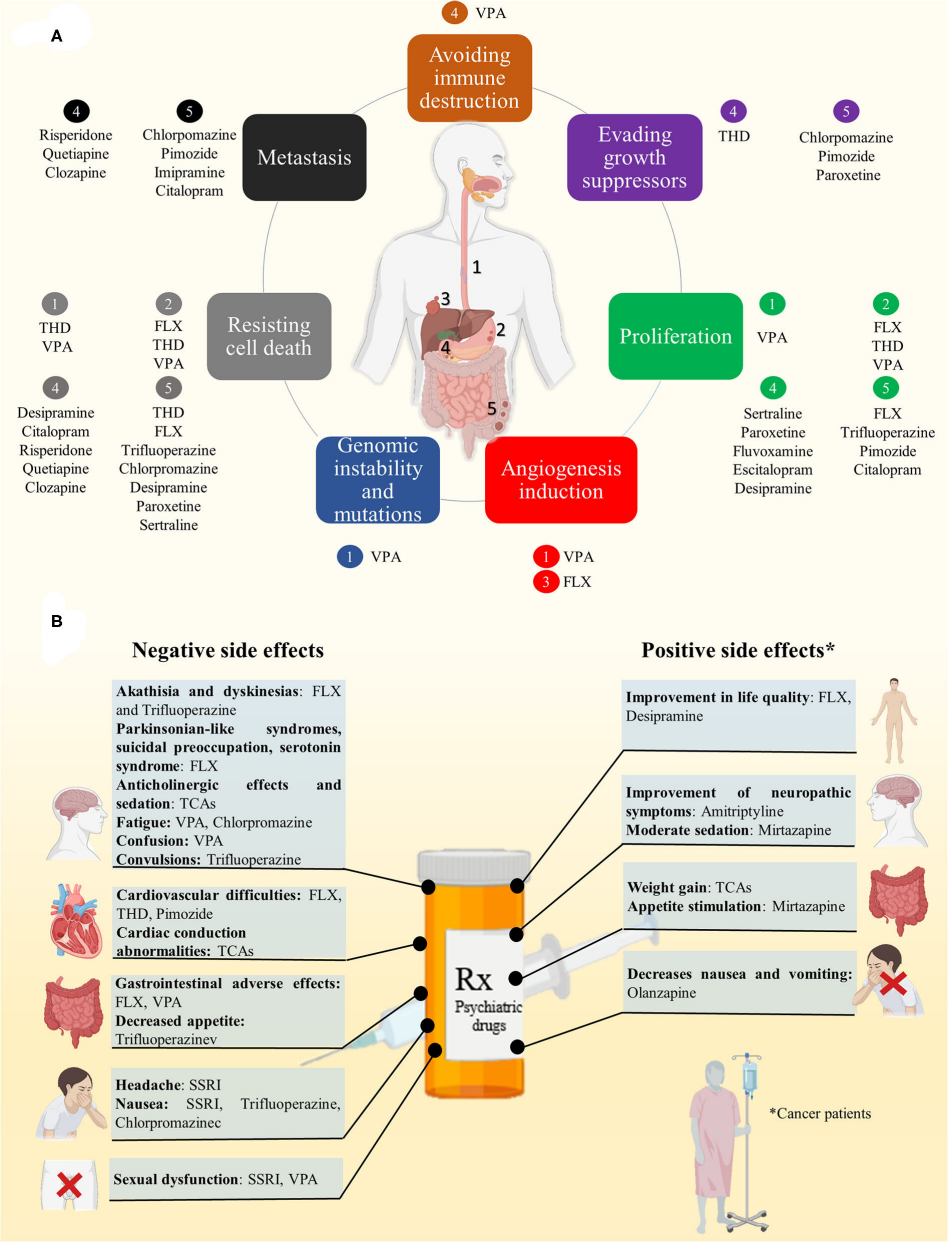
**(A)** Refocusing psychiatric drugs in GICs. PDs have shown inhibitory potential on carcinogenic processes, such as, growth, proliferation, angiogenesis, metastasis and cell survival on esophageal (1), gastric (2), liver (3), pancreatic (4), and colorectal cancer (5). **(B)** PDs exhibit positive and negative side effects that could affect the general state of patients and need to be considered.

The repurposing potential of PDs has ignited interest in further exploration for treating GICs. The aim of this review is to compile the most recent and relevant information about how psychiatric drugs have been redirected due to their inhibitory potential on carcinogenic processes, such as, growth, proliferation, metastasis and cell survival in GICs (Figure 1A).

## Refocusing Psychiatric Drugs on GICS

### Esophageal Cancer

Thioridazine (THD), an antagonist of the dopamine receptor D2, is a potent anti-anxiety and anti-psychotic that in combination with radiotherapy promotes G0/G1 phase cell cycle arrest by CDK4 and cyclinD1 downregulation on the esophageal squamous cell carcinoma (ESCC) ECA-109 and TE-1 cell lines. It also induces apoptosis upregulating cleaved caspase-3 and 9 as well as Bax, Bak, and p53, and decreasing Bcl-2 and Bcl-xL expression. Besides, this treatment inhibits PI3K-AK-mTOR pathway. On xenograft mouse model, THD/radiotherapy reduces ESCC tumor growth (9).

Valproic acid (VPA), an anti-epileptic (10), inhibits radiation-induced double-strand break (DSB) reparation by non-homologous end joining (NHEJ) suppression, specifically through Ku70 acetylation on the human ESCC KES, TE9 and TE11 cell lines. Also, VPA after irradiation prolongs H2AX phosphorylation (γH2AX) levels that result on foci formation detection (11). In addition, VPA inhibits viability in a dose-dependent manner leading to apoptosis, and radiation-induced cytotoxicity by chromatin decondensation with H3 and H4 hyperacetylation/Rad51 downregulation on ESCC TE9, TE10, TE11, and TE14 cell lines (12). Interestingly, VPA leads to Epithelial Mesenchymal Transition (EMT) reversal induced by irradiation on ESCC TE9 cells, attenuating cell invasion, and migration (13).

### Gastric Cancer

Fluoxetine (FLX) is a selective serotonin reuptake inhibitor (SSRI) to treat depression and panic disorder (14). In the human Gastric Adenocarcinoma cell line AGS, FLX inhibits proliferation in a dose-dependent manner. Moreover, FLX induces apoptosis increasing activated caspases, cleaved PARP, and promoting ROS production. Interestingly, FLX-treated AGS cells exhibit upregulation of CHOP (an endoplasmic reticulum stress marker) and its inhibition suppress partially apoptotic effects of FLX through death receptor 5 (DR5), cleaved caspase 3, and cleaved PARP downregulation (15). Besides, FLX induces both, protective autophagosome formation and apoptosis simultaneously *in vitro*. However, autophagy inhibition enhances FLX-induced apoptosis through decrease p-Akt and increase DR4 and DR5 expression (16). Moreover, it has been reported an improved anti-proliferative effect due to FLX/paclitaxel combination on GACs (AGS cell line). In addition, FLX/paclitaxel promotes cell death by early apoptotic and late apoptotic cell as well as necroptosis (17).

THD has cytotoxic effect on Gastric cancer (GC) cell lines NCI-N87 and AGS, inhibiting colony formation ability, and induces nuclear fragmentation and apoptosis in a caspase-dependent manner through downregulation of caspase-9, caspase-8, and caspase-3 precursors. Also, *in vivo* experiments using BALB/c nude mice showed an inhibition of tumor growth using pre-treated GC cells with THD (18).

VPA-treated AGS and SGC-7901 cells repress HDAC1/2 (histone deacetylase) activity and induces autophagy driving to apoptosis through HDAC1/PTEN/Akt signaling pathway inhibition, as well as Bcl-2 and Beclin-1 alterations. VPA treatment on xenograft models have shown significant inhibitory effect on cell growth via autophagy/apoptosis (19). VPA affects cell viability on GC OCUM-2MD3 cells, a derived cell line from human scirrhous GC. Besides, VPA increases acetyl-histone H3, acetyl-a-tubulin and p21WAF1 expression as well as p27, caspase 3, and caspase 9 upregulation. Furthermore, VPA downregulates bcl-2, cyclin D1, and survivin. *In vivo* experiments using xenograft models with previously VPA-treated cells inhibits tumor volume and apoptosis (20). Additionally, VPA inhibits proliferation by cell cycle arrest in G1 phase. Apoptosis activation induced by VPA is conducted mainly via intrinsic pathway through Caspase 9 and Caspase 3 activation and partially via extrinsic pathway by caspase-8 activation in BGC-823 gastric carcinoma cells. In addition, P21^Waf/cip1^, Mad1 upregulation and Cyclin A, c-Myc downregulation result in cell cycle arrest in G1 phase (21).

### Colorectal Cancer

Trifluoperazine, an anti-psychotic, inhibits cell viability and proliferation, induces apoptosis and promotes cell arrest at G0/G1 phase by repressing CDK2, CDK4, cyclin D1, cyclin E through the increasing p27 expression on HCT116. Also, this effect has a synergized effect with 5-fluorouracil and Oxaliplatin in CT26 and HCT116 colorectal cancer cells (22, 23).

THD treatment exhibits anti-tumor effect on CSCs EpCAM^+^/CD44^+^ subpopulations isolated from HCT116 through apoptosis induction via mitochondrial pathway since THD promotes upregulation of caspase-3 and Bax and downregulation of Bcl-2 suppressing proliferation and invasion (24).

Chlorpromazine reduces psychotic symptoms and has a potent anti-tumor activity (25–27). In CRC cell lines, such as, HCT116, LoVo, HT15, and HT29, chlorpromazine can suppresses cell growth, activates apoptosis by p53 acetylation and induces SIRT1 degradation acting as a cell-cycle regulator (28). Another anti-psychotic, Pimozide, dissociates the interaction between ARPC2 and vinculin suppressing focal adhesion, which blocks migration in DLD-1 CRC cells (29). Besides, Pimozide shows anti-proliferative features, suppresses migration in a dose-dependent manner, and increases N-cadherin, vimentin, and Snail expression as well as inhibits Wnt/β-catenin signaling pathway in HCT116 and SW480 cells. Also, inhibits tumor growth in xenografts mouse model (30).

Regarding tricyclic anti-depressants (TCAs), desipramine promotes apoptosis and modulates cell-cycle arrest at G0/G1-phase reducing a dose-dependent S-phase proportion cells in HT29 CRC cell line (31). Besides, FLX treatment target early colon carcinogenesis due to its anti-proliferative effect by affecting ROS production, DNA damage, cell viability, apoptosis, and cell-cycle in HT29 cell line. Besides, using C57BL/6 mice exposed to the carcinogen N-methyl-N'-nitro-N-nitrosoguanidine (MNNG) was demonstrated that FLX affects proliferation mainly in epithelial and stromal areas accompanied by reduction of VEGF expression and number of cells with angiogenic potential, such as CD133^+^, CD34^+^, and CD31^+^ (32).

Imipramine also compromises HCT116 cells viability at low concentrations and inhibits lamellipodium formation, cell migration and invasion by affecting fascin1 (33). On the other hand, has been reported that paroxetine reduces cell viability and increases apoptosis; it also inhibits colony and 3D spheroid formation in HCT116 and HT29 cell lines. Probably, paroxetine can act by the inhibition of MET and ERBB3 which are two major receptor tyrosine kinases, leading to the suppression of AKT, ERK and p38 activation and induction of JNK and caspase-3 pathways. Also, *in vivo* assays using athymic nude mice treated with paroxetine showed tumor growth suppression (34).

In the case of the SSRI Citalopram, it inhibits migration in HT29 and HCT116 cell lines. Besides, it has anti-proliferative effect on xenograft mouse model as well as in orthotopic mouse model, acting against metastatic progression (35). Another SSRI, Sertraline, arrest cells at the G0/G1 stage and stimulates DNA fragmentation in a dose-dependent manner and at low concentrations promotes apoptosis by increasing caspase-3 activation and c-Jun expression as well as a decrease in the anti-apoptotic protein Bcl-2 (36).

The dopamine inhibitor hydrochloride (IND), the dopamine receptor antagonists chlorpromazine hydrochloride (CPZ), and fluphenazine dihydrochloride (FPZ) are able to increase LC3-II in a dose- and time-dependent manner in HCT116 cells. Moreover, FPZ induces autophagy by mTOR signaling inhibition, meanwhile IND and CPZ induces autophagy in an mTOR-independent manner. However, in this context autophagy does not stimulates apoptosis, rather offers beneficial effects for cell survival in HCT116 treated with IND, CPZ and FPZ (37).

### Liver Cancer

SSRIs including FLX, sertraline, paroxetine, citalopram, escitalopram, and fluvoxamine are associated with a lower risk of hepatocellular carcinoma (HCC) in a dose-dependent manner (38). Also, sertraline, paroxetine, fluvoxamine, and escitalopram have shown significant decrease of cell viability in HCC, being sertraline the most efficient to induce caspase-3/7 activity in HepG2 cells (39). Another anti-depressant, desipramine reduces cell viability by increasing ROS production, inhibits MMP activity in Hep3B cells. Besides, it is able to induce apoptosis through MAPKs activation (ERK 1/2, JNK, and p38), companied of intracellular Ca^2+^ levels increase (40). In addition, citalopram activates NF-kB and is a potent apoptosis-inductor since increases mitochondrial Bax levels, decreases in Bcl2 levels, increases ROS and releases cytochrome c release in HepG2 cells (41).

The use of risperidone, quetiapine, and clozapine, have shown reduction of cell viability, cell proliferation, invasion, and induction of apoptosis *in vitro*, in Huh7, and HepG2 cells (42). Meanwhile, several HCC cell lines treated with the anti-psychotic pimozide exhibit inhibition of cell proliferation and sphere formation through G0/G1 phase cell cycle arrest as well as cell migration. In addition, it suppresses STAT3 signaling. Besides, pimozide inhibits stemness properties of HCC stem-like cells as colony formation, sphere formation, migration and has the capacity to reverses the stem phenotype. *In vivo*, pimozide reduces the tumor burden in the nude mice xenograft model (43) and affects cell viability on Hep3B and HepG2 cells by inhibition of·Wnt/β-catenin signaling and EpCAM gene and protein expression (44).

### Pancreatic Cancer

Mirtazapine is a novel noradrenergic and specific serotonergic anti-depressant (NaSSA) (45) that helps during gemcitabine-induced mild cachexia in pancreatic tumor-induced on mice (46). Olanzapine, an anti-psychotic, inhibits survivin expression on pancreatic CSCs which results on chemosensibilization to 5-fluorouracil, gemcitabine and cisplatin (47) (Table 1). On the other hand, 5-HT uptake promotes activation of Rac1 resulting in trans-differentiation of primary acinar cells into acinar-to-ductal metaplasia (ADM), a determinant step to Pancreatic Ductal Adenocarcinoma (PDAC). However, FLX inhibits to Slc6a4, a 5-HT transporter Sert, reducing ADM formation both *in vitro* and *in vivo* on mice model. Besides, FLX treatment inhibits proliferation, tumor microenvironment and alters lipid metabolism of several PDAC cell lines (49).

**Table 1:** Synergistic effects of PDs and chemotherapeutic agents in GICs.

**Psychiatric drug**	**Chemotherapy drug**	**Effect**	**References**
Trifluoperazine	5-Fluorouracil Oxaliplatin	Viability inhibition of CT26 and HCT116 colorectal cancer cells	(23)
Olanzapine	5-Fluorouracil Gemcitabine Cisplatin	Cell death in PANC-1 cell after chemosensitization by survivin downregulation	(47)
VPA	Gemcitabine	Low-dose VPA significantly enhances cytotoxicity of pancreatic cancer cells to gemcitabine	(48)
Fluoxetine	Paclitaxel	Fluoxetine-paclitaxel combination exhibits anti-proliferative effect on gastric adeno carcinoma cells by G2/M arrest and increased events in the sub G0/G1 phase	(17)

VPA inhibits cell proliferation and cell attachment to the endothelium on DanG cells and enhances β1 integrin subunits expression such as α4, α5, and α6 (50). Besides, VPA inhibits cell survival and induces apoptosis via EGFR/ErbB2/ErbB3 downregulation on HPAF-II, MPanc96 PC cells and inhibits tumor growth on xenograft models (51). Moreover, VPA increases susceptibility of PANC-1, MIA PaCa-2, and BxPC-3 PC cell lines to NK cell-mediated lysis both *in vitro* and in *vivo* (xenografts in NOD/SCID mice) by MICA and MICB upregulation in a dependent mechanism of PI3K/Akt signaling pathway (52). Interestingly, high-dose VPA in combination with gemcitabine has a synergic effect enhancing the sensitivity of PANC-1 and Patu8988 PC cell lines to gemcitabine, meanwhile low-dose of VPA potentiates migration, invasion, and promotes ROS production, activating AKT that induces STAT3/Bmi1 signaling cascade activation as well as migration and invasion of PC cells induced by gemcitabine. However, high-dose VPA stimulates ROS accumulation promoting p38 activation and suppressing STAT3/Bmi1 activation (53). Combination of VPA with PEG-IFNα inhibits cell proliferation on BxPC3 PC cell line by caspase-3/7 activity promotion. In addition, the combination sensibilizes BxPC3 and SUIT-2 PC cells to interferon-α, promoting IFN-related genes expression, IFNAR1, IFNAR2, and IRF8 (54).

In the case of the anti-psychotic drugs penfluridol, pimozide, fluspirilene, and promethazine have an antagonist effect on proliferation and induce autophagy by LC3II and p62 upregulation in Panc-1, and MiaPaCa-2 human PDAC cell lines. Also, penfluridol suppress colony and spheroid formation and decreases PRL-induced JAK2 signaling by binding to PRLR. *In vivo*, using NSG mice, penfluridol have shown tumor growth suppression (55). Additionally, penfluridol shows cytotoxic effects as well as autophagy-mediated apoptosis in the pancreatic cancer Panc-1, AsPC-1, and BxPC-3 cell lines and is able to suppress subcutaneously implanted pancreatic tumor growth through autophagy induction on athymic nude mice. Finally, Panc-1 cells implanted into the pancreas of mice shows that penfluridol increases p62 and LC3B as well as cleavage caspase3 and PARP (56).

## Clinical Implications and Side Effects

PDs exhibit side effects that need to be considered (Figure 1B):

- SSRI anti-depressants: headache, nausea, and sexual dysfunction, as well as several cytochrome P450 drug interaction (57).
- FLX: cardiovascular difficulties, akathisia, dyskinesias, parkinsonian-like syndromes, suicidal preoccupation, “serotonin syndrome” (58), gastrointestinal adverse effects compared to other anti-depressants (59).
- FLX and Desipramine offer benefits on quality of life issues in patients with advanced cancer (60).
- TCAs: cardiac conduction abnormalities, anti-cholinergic effects, weight gain and sedation. Specifically, Amitriptyline and Imipramine induce sedation, anti-cholinergic effects, weight gain, sexual dysfunction, sedation, and cardiac effects (61). However, weight gain associated to TCAs can be a well feature on cancer patients. Besides, low doses of Amitriptyline improve several neuropathic symptoms which result in a best quality of life on cancer patient (62).
- VPA: fatigue, confusion, neuroconstipation, and somnolence (63).
- Trifluoperazine: decreased appetite and induces nausea, insomnia, akathisia, dyskinesias, skin disorders, and tremors including convulsions, as well as, hyperprolactinaemia and galacthorrea (64–67).
- Chlorpromazine: nausea, drowsiness, and tiredness (68).
- THD and Pimozide: cardiac effects (69).
- Mirtazapine: can induces moderate sedation and appetite stimulation that can enhances patients' wellness (70).
- Olanzapine: decrease chemotherapy side effects as nausea and vomiting in advanced cancer patients (71).

## Concluding Remarks

GICs still rank among the leading causes of cancer related death because of late-stage detection and poor survival following metastasis. Treatments depend on multiple factors and constantly fail. Psychiatric drugs (PDs) are promising as a new generation of cancer chemotherapies due to their anti-neoplastic properties in a plethora of cancer types.

Drug repurposing potential of PDs has been studied for the treatment of GICs founding that these drugs can modulate several cellular processes as cell viability, cell growth, proliferation (cell cycle arrest), apoptosis, autophagy, ROS production, and DNA fragmentation. Besides, PDs can inhibit neovascularization, invasion, and metastasis and effects can be in a dose-dependent manner, such as, VPA, FLX, Pimozide, Desipramine, and Sertraline. Moreover, PDs can induce chemosensibilization as Olanzapine combined with 5-fluorouracil, gemcitabine, and cisplatin induce cell death. In addition, low-dose of VPA enhances cytotoxicity to gemcitabine. FLX also shows anti-proliferative effect in combination with paclitaxel, while, trifluoroperazine exerts viability inhibition when combined with 5-fluorouracil and oxaliplatin.

The modulation of several processes by PDs can be through activation or inhibition of multiple pathways such as HDAC1/PTEN/Akt, EGFR/ErbB2/ErbB3, and PI3K/Akt (VPA); PI3K-AK-mTOR, HDAC1/PTEN/Akt (THD); Wnt/β-catenin (pimozide).

Finally, PDs as repurposed drugs, could be more affordable for patients and could also reduce costs for drugs developers. PDs might improve cancer treatments as well as life quality of patients in long therm. Nevertheless, further investigations and clinical trials are needed to elucidate all molecular mechanisms involved on anti-cancer activities of PDs as well as negative side effects on patients.

## Author Contributions

MA-F, MA-M, MB, EL-V, and RR-P conceived and designed the content of this review and wrote the paper. CL-C contributed to the final version of the manuscript. All authors contributed to the article and approved the submitted version.

## Conflict of Interest

The authors declare that the research was conducted in the absence of any commercial or financial relationships that could be construed as a potential conflict of interest.
